# An Intuitive Approach to Understanding the Attributable Fraction of Disease Due to a Risk Factor: The Case of Smoking

**DOI:** 10.3390/ijerph10072932

**Published:** 2013-07-16

**Authors:** Laura Rosen

**Affiliations:** School of Public Health, Sackler Faculty of Medicine, Tel Aviv University, PO Box 39040, Ramat Aviv 69978, Israel; E-Mail: rosenl@post.tau.ac.il

**Keywords:** attributable fraction, burden of disease, smoking-attributable mortality (SAM), tobacco

## Abstract

The health damage from tobacco use has been studied intensively, yet quantifying the precise burden of disease and death due to smoking is a complex problem, and consequently open to manipulation by interested parties. The goals of this paper are to clearly communicate the concept of the attributable fraction (AF), *i.e*., the proportion of disease in a population which can be attributed to a risk factor, and to understand the relationship between the AF, the prevalence of exposure in a population, and the relative risk of disease given the exposure. The current approach to calculating the AF is summarized. An intuitive formula is proposed, with accompanying graphical illumination. For diseases caused by smoking, the AF of disease due to smoking increases with the prevalence of smoking and with the relative risk of disease due to smoking. The proposed method has the potential to help health professionals and decision makers understand the concept of the burden of disease due to smoking or other lifestyle, environmental, and occupational factors, in the context of public health importance. This will aid sound decision-making in public health policy.

## 1. Introduction

Tobacco, the leading cause of preventable death for both men and women (p. i in [1]) is estimated to have killed one hundred million people in the last century; up to a billion may die due to smoking in the current century [2]. As with obesity, and environmental and occupational exposures, knowledge of the magnitude of the burden due to tobacco use has been the stimulus for global, national, and local initiatives, yet estimating the extent of damage from active and passive smoking is complex. Disagreements may stem from purely scientific concerns or as a result of powerful vested interests, such as the tobacco industry [3].

The root of the problem in quantifying the precise amount of mortality and morbidity caused by smoking lies in assessing whether a given death was caused by a particular behavior or condition. Even reasons for deaths due to such obvious causes as traffic accidents, drownings, or febrile pneumonia may be difficult to ascertain. Certainty that any individual illness or death is directly due to tobacco use or secondhand smoke exposure is far more difficult. Numerous methods have been suggested for assessing the magnitude of the burden (pp. 878–880 in [4]); most are based on estimating the harm from decades of observation of smokers and nonsmokers, and use of mathematical models.

The goals of this paper are to clearly communicate the concept of the attributable fraction (AF), the proportion of disease in a population which can be attributed to a risk factor, and to understand the relationship between the AF, prevalence of exposure in a population, and relative risk of disease given the exposure.

### 1.1. Attributable Fraction

The quantification of the burden of disease or death due to a specified exposure—in this case active tobacco smoking—is generally done in three steps (p. 878 in [4]).

The first step involves identification of diseases which are caused by smoking, and quantification of the increased risk to an individual due to smoking. This is generally done by using data from large prospective cohort studies which document behaviors, disease, and mortality over decades, such as the British Physician’s Study [5], and Cancer Prevention Studies I and II [6].

The second step is to quantify the proportion of disease in a population that can be attributed to a specified exposure. This is done through use of the attributable fraction (AF), which is also known as the excess fraction, the population attributable risk, the population attributable risk proportion, the etiologic fraction, and the incidence density fraction [4,7]. The AF is the central concept in the estimation of burden for behaviors and exposures [4]. In the case of smoking, the term “smoking-attributable fraction (SAF)” is often used instead of “attributable fraction”.

The third step involves summation of the number of cases due to smoking across all smoking-related illnesses.

### 1.2. Original Formula for the Attributable Fraction

A formula for the AF was first proposed by Levin in 1953 [8]. The beauty of Levin’s formula, still the most popular, is that it is easy to calculate, and is dependent on generally accessible estimates regarding 1—the underlying prevalence of the risk factor in the population, and 2—the relative risk of developing the disease among those with, *versus* those without, the risk factor. Levin’s original formula, published in 1953, is:

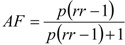
(1)
where:

- p = the underlying prevalence of the risk factor in the population (for example, the prevalence of smoking in the population), and
- RR (Relative Risk): Risk of contracting a disease in an exposed population (e.g., smokers) divided by the risk of contracting the disease in an unexposed population (e.g., nonsmokers)

However, in part because the underlying meaning of this formulation of the AF is not intuitively obvious, the concept of the AF may be poorly understood.

## 2. Materials and Method

A simple formula for the AF, accompanied by a graphical presentation, is proposed, followed by worked examples of AF calculation in countries with different smoking rates. A proof of the algebraic equivalence of the proposed formula to the accepted formulation by Levin is presented in Appendix I. The mathematical relationships which determine the size of the attributable fractions are explored graphically. This method is then compared with other existing approaches.

## 3. Results

### 3.1. Proposed Formula for the Attributable Fraction

The proposed formula is:

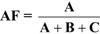
(2)
where:

**A** = Number of smokers in the population who contract a particular disease, Disease X, due to their smoking habit
**B** = Number of smokers in the population who contract a particular disease, Disease X, but not due to their smoking habit
**C** = Number of nonsmokers in the population who contract Disease X.

If we let T = the total number of people who contract Disease X, then the formula may be re-expressed as:

AF = A / T
(3)

### 3.2. Graphical Illustration of Proposed Formula

**Figure 1:**
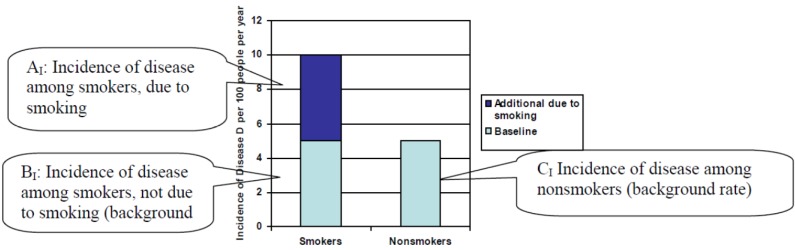
Incidence of disease among smokers and nonsmokers.

The formula is illustrated through use of two graphs. The first, Figure 1, describes the incidence (that is, the number of new cases which occur) of a hypothetical disease, Disease X, in an exposed population and an unexposed population. In this case, smokers form the exposed population, and nonsmokers form the unexposed population (A similar graph has been presented previously [9]). For our hypothetical example, the following are assumed to be true: the incidence of Disease X among nonsmokers is 5 per 100 per year (C_I_) and the incidence of Disease X among smokers is 10 per 100 per year. Half of the incidence among smokers (B_I_) is due to the baseline incidence of Disease X, while half of the incidence among smokers (A_I_) is due to the additional risk for Disease X taken on by smokers.

In this example, smokers take on an additional risk of 5 cases per 100 people per year. Of every two smokers who contract Disease X, one of them contracts it because of their smoking habit. Smokers are twice as likely to contract Disease X as are nonsmokers.

**Figure 2:**
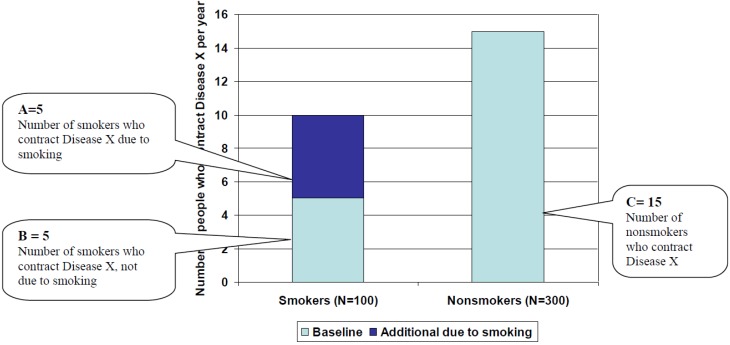
Number of smokers and nonsmokers who contract disease.

Figure 2 shows the **numbers** (as opposed to the **incidence**) of smokers and nonsmokers who contract Disease X. We assume that there are 400 people in the population, and that the proportion of smokers in the population is 25%. We assume that, as in Figure 1, smokers have an incidence of Disease X of 10 per 100 people per year, while nonsmokers have an incidence of Disease X of 5 per 100 people per year.

**A** represents the **number** of smokers who contract Disease X due to smoking:

*A = Incidence in smokers due to smoking × number of smokers = 5/100 × 100 smokers = 5*

**B** represents the number of smokers who contract Disease X, unrelated to smoking:

*B = Incidence among smokers not due to smoking (equal to incidence of disease in nonsmokers) × number of smokers = 5/100 × 100 = 5*

**C** represents the number of nonsmokers who contract Disease X, unrelated to smoking:

*C = Incidence in nonsmokers × number of nonsmokers = 5/100 × 300 = 15*

The Attributable Fraction (AF) is defined as the **number** of people who contract Disease X as a result of smoking, divided by the total number of people who contract Disease X. Therefore: 
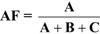
. As pictured in Figure 2, AF = 5/25 = 20%.

### 3.3. Relationship between the Attributable Fraction, the Relative Risk, and the Proportion of Smokers in the Population

The primary contribution of the AF is to help policy makers prioritize [10]. High or even moderate attributable fractions may provide the impetus necessary to act, depending on the frequency and severity of the condition in the population. How the magnitude of the AF varies with the relative risk and the prevalence of smoking is demonstrated below using a set of illustrative examples and a graphical presentation.

### 3.4. Illustrative Examples

We illustrate properties of the AF using a hypothetical example based on the prevalence of male smoking from four countries: Cameroon, Canada, Japan, and Greece (Respectively: 9.9%, 19.0%, 44.3%, 63.6%) [11]. For simplicity, we assume that all male smokers smoke between 15–24 cigarettes per day. Though any N would produce the same value of the AF, for the purposes of demonstration, we assume a population of 1 million male residents per country. We also assume that the incidence of disease due to smoking, and relative risks of disease for smokers *versus* nonsmokers, are identical to those reported in the British Physician’s Study [5]. The data are shown in Table 1.

**Table 1:** Hypothetical burden of lung cancer and cardiovascular mortality due to smoking among males.

Disease	Incidence among nonsmokers per 1,000 men/year	Incidence among smokers (15–24 cigarettes per day) per 1,000 men/year	Assumed Relative Risk of mortality among smokers (15–24 cigarettes per day)	Male Smoking Prevalence	A	B	C	Hypothetical Attributable Fraction
Lung cancer	0.17	2.33	13.7	9.9% ^1^	214	17	153	55.7%
	0.17	2.33	13.7	19.0% ^2^	410	32	138	70.7%
	0.17	2.33	13.7	44.3% ^3^	957	75	95	84.9%
	0.17	2.33	13.7	63.6% ^4^	1,374	108	62	89.0%
Heart disease	6.19	10.07	1..6	9.9% ^1^	384	613	5,577	5.8%
	6.19	10.07	1.6	19.0% ^2^	737	1,176	5,014	10.6%
	6.19	10.07	1.6	44.3% ^3^	1,719	2,742	3,448	21.7%
	6.19	10.07	1.6	63.6% ^4^	2,468	3,937	2,253	28.5%

Notes: Under the following assumptions: 1—All male smokers smoke between 15–24 cigarettes per day; 2—Relative Risks for disease in these countries in smokers *versus* nonsmokers are identical to those found by Doll *et al.* [5]; ^1^ = Cameroon, ^2^ = Canada, ^3^ = Japan, ^4^ = Greece.

We utilize mortality data from two diseases known to be associated with smoking: lung cancer and heart disease. Data from the British Physician’s Study [5] show an underlying incidence among male lifetime nonsmokers of 0.17 per 1,000 individuals per year for lung cancer and 6.19 for ischaemic heart disease, and incidence rates for males smoking 15–24 cigarettes per day of 2.33 per 1,000 person years for lung cancer and 10.07 per 1,000 person years for ischaemic heart disease. Thus, the relative risk for male smokers who smoked 15–24 cigarettes per day was 13.7 for lung cancer and 1.6 for heart disease. A worked example of calculations for the first row in Table 1 is presented in Appendix II.

The AFs for lung cancer were 55.7%, 70.7%, 84.9%, 89.0% for Cameroon, Canada, Japan, and Greece. For heart disease the AFs were much lower: 5.8%, 10.6%, 21.7%, 28.5% for the four countries, respectively. These differences reflect the extremely high RR for lung cancer, relative to the lower RR for heart disease.

**Figure 3:**
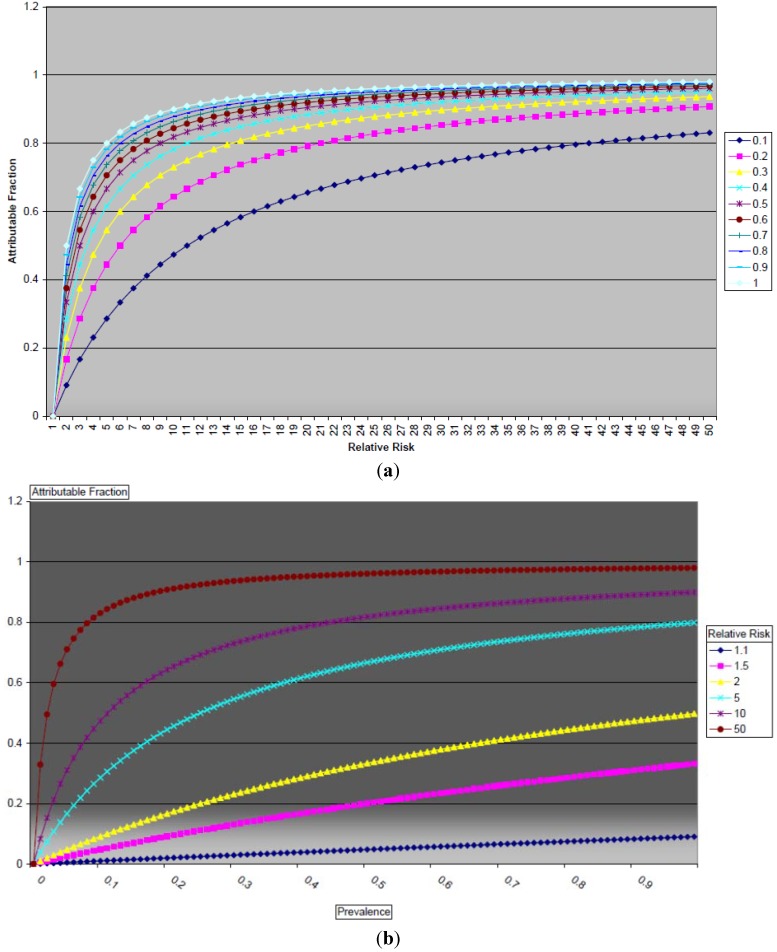
(**a**) Relationship between Attributable Fraction and relative risk of disease due to smoking as a function of smoking prevalence; (**b**) Relationship between Attributable Fraction and population smoking prevalence as a function of relative risk.

The burden of smoking on disease incidence in the population is low when the prevalence of smoking in the population is low and when the RR of disease due to exposure is low. This is the explanation for the low hypothetical AF in Cameroon for heart disease (AF = 5.8%): the prevalence of smoking in the population is low (9.9%), and the RR for heart disease is moderate (1.6). The converse is also true: when the RR is high and the prevalence is high, the AF will be high. This also explains why the greatest AF in our example occurs for lung cancer in Greece (AF = 89.0%): Greece has the highest male smoking prevalence of the countries shown in the table (63.6%), and the RR of lung cancer for smokers (15–24 cigarettes per day) is very high (13.7).

### 3.5. Mathematical Relationships between the Attributable Fraction, the Relative Risk, and the Prevalence of Smoking

In order to further explore the relationship between the AF, prevalence of smoking, and the relative risk of disease due to a risk factor, beyond the binary categories of high/low prevalence and high/low relative risk, we graphed the relationships between these three entities. Figure 3(a,b) illustrate the mathematical relationship between the AF, RR, and the prevalence of smoking in a population. The AF can take on a minimum value of 0 and a maximum value of 1. We restrict the discussion to situations in which the RR is greater than 1, that is, the exposure in question causes harm. In Figure 3a, the relationship between AF and RR is examined, with different values of p, the prevalence of smoking. For all values of p, as the RR increases, the AF increases monotonically, and approaches its maximum value of 1. In Figure 3b, the relationship between the AF and the prevalence is examined. As p approaches its maximum value of 1, the limit of the AF is the quantity (RR-1)/RR. When p = 1, the AF approaches the value of 1 as RR increases.

## 4. Discussion

The current manuscript attempts to clarify the concept of burden of disease due to tobacco use through proposing an intuitive approach to understanding the AF, and through an exploration of how the AF changes under various scenarios. This approach is not restricted to smoking, and can be used to understand the disease burden due to such factors as secondhand smoke exposure, obesity, physical inactivity, and environmental hazards. This approach adds a potentially useful tool for health communication, and may help policy makers easily conceptualize an important concept, thus avoiding the human biases which thrive in the face of uncertainty [12].

Since Levin published his original formulation for the AF, a number of alternate proposals have been made [13,14,15,16]. Bruzzi proposed a method of calculating AFs while adjusting for confounders [14]. McNulty used smoking status reports from death certificates [15]. More recently, Peto’s “indirect” method used lung cancer rates to retroactively estimate smoking prevalence [16]. Overviews of various approaches and scientific issues have been provided by, among others, Benichou [17], Steenland [18], the US Surgeon General [4] and Rockhill [7].

Most existing formulations of the AF [8,19,20] rely on the incidence of disease in the exposed and unexposed population, or on the relative risk of disease. Making the “jump” from incidence or relative risks, on the one hand, to numbers of people harmed, on the other, given an unequal balance of smokers and nonsmokers in a population and the different incidence rates applicable to each, may be challenging for some people without training as epidemiologists. The proposed approach illustrates this “jump” from incidence to actual numbers. It assumes that a causal relationship between disease and exposure has been established.

For policy makers, the AF is important for two reasons. First, it is important to understand the proportion of illness which is attributable to a specific cause. If, for example, policy makers were interested in decreasing the burden of lung cancer, the knowledge that 90% of lung cancer is attributable to smoking would help make tobacco control a priority. Second, the actual burden of a specific disease is of great interest to policy makers: a condition which is not prevalent and not serious wouldn’t garner much attention, while a condition such as heart disease, which is both prevalent and serious, is the topic of much attention. The magnitude of the AF, in combination with the frequency and severity of the assessed condition in the population, will together determine the priority a given exposure should have in improving health of populations. Northbridge previously examined the effect of size of relative risk (high, low) and population prevalence of risk factor (high, low) on the size of the attributable fraction, and showed that, for example, lung cancer, with its high RR and high smoking population prevalence, has a high public health priority [10]. In this paper, the mathematical relationship between relative risk, population prevalence, and the AF has been explored, and includes a complete, illustrated presentation of the relationships.

Important new advances in the science of studying disease burden have occurred over the course of the past decades. The massive Global Burden of Disease Project [21,22], which investigates the global burden of disease using the disability-adjusted life year, serves as a major input to policy-making on a global scale. A recent analysis by this group showed tobacco (smoking and exposure) to be one of the top three risk factors for global disease and disability [23]. One interesting approach is counter-factual analysis, which compares existing estimates of burden with those based on a different exposure profile in the population [24].

## 5. Conclusions

The proposed approach should be helpful to decision makers, clinicians, and students of medicine and public health in understanding the concept of burden of disease due to smoking and, by extension, other risk factors and exposures. A clear understanding of these concepts should aid sound decision-making in the areas of public health and clinical policy.

## List of abbreviations

AF: Attributable Fraction
CDC: US Centers for Disease Control
SAMMEC: Smoking attributable mortality, morbidity, and economic costs
p: Underlying prevalence of a risk factor in a population
RR: Relative Risk: Risk of contracting a disease in an exposed population divided by the risk of contracting the disease in an unexposed population
A: Number of smokers who contract a particular disease—“Disease X”—due to their smoking habit
B: Number of smokers who contract a disease, unrelated to their smoking habit
C: Number of nonsmokers who contract a given disease (unrelated to smoking)

## Appendix I: Proof

We begin with Levin’s original formula:

We first present an empirical example, using the numbers previously used in the text and the graphs. In that example, the incidence of Disease X among smokers was 10% while the incidence of Disease X among nonsmokers was 5%; thus the relative risk of disease for smokers *versus* non-smokers was 2.0. The prevalence of smoking in the population was 25%. Thus, according to Levin’s formula, AF = 25% × (2 − 1)/[25% × (2 − 1) + 1] = ¼ ÷ 5/4 = 20%.

Let

I_S_ = the incidence of disease among smokers,

I_N_ = the incidence of disease among nonsmokers

p = proportion of smokers in the population Assume rr refers to the relative risk of disease incidence.

N = number of people in population

Then

If we set

A = Np(IS – IN)

B = NpIN

C = N(1 – p)IN

Then this is equivalent to our proposed formula, A/(A + B + C).

## Appendix II: Worked Example

As an example, detailed calculations for the first row in Table 1 (Cameroon, lung cancer) are presented. To calculate A, it is necessary to know the number of smokers and the incidence of lung cancer due to smoking. Assuming a population of 1 million with a prevalence of 9.9%, the number of smokers is 99,000. The incidence due to smoking is the difference between the incidence among smokers and the incidence among nonsmokers e.g., 2.33/1,000 person-years—0.17/1,000 person-years = 2.16/1,000 person-years. Therefore, A = (2.16/1,000) × 99,000 = 213.8.

In order to calculate B, it is necessary to know the number of smokers and the incidence of lung cancer among nonsmokers. The number of smokers is 99,000, and the incidence of lung cancer among nonsmokers is 0.17/1,000 person-years. Therefore B = 0.17/1,000 person-years × 99,000 = 16.8.

To calculate C, it is necessary to know the incidence in nonsmokers (0.17/1000 person-years) and the number of nonsmokers (90.1% × 1,000,000 = 901,000). C = 0.17/1,000 × 901,000 = 153.2.

The AF is therefore A = (A + B + C) = 55.7%
